# PEGylation of Superparamagnetic Iron Oxide Nanoparticles with Self-Organizing Polyacrylate-PEG Brushes for Contrast Enhancement in MRI Diagnosis

**DOI:** 10.3390/nano8100776

**Published:** 2018-09-29

**Authors:** Erzsébet Illés, Márta Szekeres, Ildikó Y. Tóth, Katalin Farkas, Imre Földesi, Ákos Szabó, Béla Iván, Etelka Tombácz

**Affiliations:** 1 Department of Physical Chemistry and Materials Science, University of Szeged, Aradi Vt. 1, H-6720 Szeged, Hungary; szekeres@chem.u-szeged.hu (M.S.); Ildiko.Toth@chem.u-szeged.hu (I.Y.T.); 2 Department of Laboratory Medicine, University of Szeged, Semmelweis u. 6, H-6720 Szeged, Hungary; lokine.farkas.katalin@med.u-szeged.hu (K.F.); foldesi.imre@med.u-szeged.hu (I.F.); 3 Polymer Chemistry Research Group, Institute of Materials and Environmental Chemistry, Research Centre for Natural Sciences, Hungarian Academy of Sciences, P.O. Box 286, H-1519 Budapest, Hungary; szabo.akos@ttk.mta.hu (Á.S.); ivan.bela@ttk.mta.hu (B.I.); 4 Department of Food Engineering, University of Szeged, Moszkvai krt. 5-7, H-6725 Szeged, Hungary

**Keywords:** superparamagnetic nanoparticles, PEG coating, core–shell nanoparticles, blood compatibility, colloidal stability, MRI contrast agents

## Abstract

For biomedical applications, superparamagnetic nanoparticles (MNPs) have to be coated with a stealth layer that provides colloidal stability in biological media, long enough persistence and circulation times for reaching the expected medical aims, and anchor sites for further attachment of bioactive agents. One of such stealth molecules designed and synthesized by us, poly(polyethylene glycol methacrylate-*co*-acrylic acid) referred to as P(PEGMA-AA), was demonstrated to make MNPs reasonably resistant to cell internalization, and be an excellent candidate for magnetic hyperthermia treatments in addition to possessing the necessary colloidal stability under physiological conditions (Illés et al. J. Magn. Magn. Mater. 2018, 451, 710–720). In the present work, we elaborated on the molecular background of the formation of the P(PEGMA-AA)-coated MNPs, and of their remarkable colloidal stability and salt tolerance by using potentiometric acid–base titration, adsorption isotherm determination, infrared spectroscopy (FT-IR ATR), dynamic light scattering, and electrokinetic potential determination methods. The P(PEGMA-AA)@MNPs have excellent blood compatibility as demonstrated in blood sedimentation, smears, and white blood cell viability experiments. In addition, blood serum proteins formed a protein corona, protecting the particles against aggregation (found in dynamic light scattering and electrokinetic potential measurements). Our novel particles also proved to be promising candidates for MRI diagnosis, exhibiting one of the highest values of *r*2 relaxivity (451 mM^−1^s^−1^) found in literature.

## 1. Introduction

Recent advancements in nanomedicine raise the expectations towards its translational medical impact [1,2,3]. Fabrication of new types of nanoparticles, on the one hand, and the intention from the side of medical science to pretreat corresponding tissues for better exploitation of nanodrugs, on the other, are new trends in the multidisciplinary approach of nanomedicine. Most of the nanoparticles are intended to be applied as anticancer agents either separately in the fields of diagnosis and therapy, or in combined theranostics [4,5,6,7,8].

Magnetic properties of some nanoparticles are widely exploited in MRI contrast enhancement, magnetic targeting, and magnetic heating [9]. For biocompatibility and bioapplicability reasons, the nanoparticles are coated by a stealth layer, which transforms the bio/nano interface so that the particles are tolerated by the biological media. Moreover, it possibly contains the bioactive agents for healing, and obeys the property of releasing the drugs at the target with required pharmacokinetics [10].

PEG (polyethylene glycol) coatings on SPIONs (superparamagnetic iron oxide nanoparticles, abbreviated in this paper as MNPs, magnetite nanoparticles) reduce the non-specific adsorption of blood proteins (among them, opsonins), and the resulting biologically passive surface protects the particles from subsequent adhesion of phagocytes or inflammatory cells [11,12]. PEG stealth layers on liposomes have been shown to reduce the nanoparticle uptake by microphages of RES (reticuloendothelial system) [12,13]. It has also been proven that charge and hydrophobic/hydrophilic property of the surface of nanoparticles influence, basically, their interaction with proteins, other biomolecules, and cell membranes [14]. Highly negatively charged and hydrophobic surfaces are the most prone to adsorb proteins, and promote their conformational changes in the adsorbed state [15,16,17,18]. Although proteins can bind to PEG coatings as well, they most likely retain natural conformation, and the recognition systems fail to identify the underlying nanoparticles as foreign bodies. This type of mechanism could explain, for example, the results of Price et al. [12] that PEG-modification of liposomes did not decrease the adsorption of blood plasma components, but prolonged, significantly, the nanoparticle circulation time. Thus, PEGs make an excellent coating for long circulating nanoparticles needed in MRI and other theranostic applications [19,20,21,22]. For therapeutic aims, specific bioactive moieties should be incorporated additionally in the coating layer. Nevertheless, the physicochemical behavior of the core–shell nanocarriers remains of primary importance [16].

The antifouling capability of PEGylated surfaces is strongly affected by the structure of the PEG layer, in addition to its molecular weight and interfacial density [17,23,24,25,26,27,28,29]. It has been found that PEG brushes enhance protein repellency more efficiently than linear PEGs [23]. The packing density of stealth layers and, consequently, their durability, can be increased by applying either covalent bonding strategies or multisite-binding physical adsorption [26,30]. Chemical anchoring processes demand harsh conditions for creating high probability covalent binding, separating the reaction products from catalyzers, eluting possible byproducts, and the non-aqueous medium. For biomedical purposes, we prefer mild conditions for surface modification. Thus, we exploited the self-organizing properties of the stealth polyelectrolyte, leading to its multisite-binding physical adsorption at the MNP surface. We applied this method to one of our newest candidates for surface modification of superparamagnetic (magnetite) nanoparticles for biomedical applications, which is a brush-like PEG copolymer, P(PEGMA-*co*-AA), designated here as P(PEGMA-AA). The PEG chains of the PEGMA units are brush-like, depending on the polymer backbone (see the structure in Table 1). In a previous publication [31], we reported on the synthesis of P(PEGMA-*co*-AA), the basic colloidal and magnetic properties of the core–shell P(PEGMA-AA)@MNPs, and their general biocompatibility and magnetic hyperthermia applicability. In the present paper, we study the self-assembly of P(PEGMA-AA) on the MNPs surface at a molecular level (using potentiometric acid–base titration, polyelectrolyte binding experiments, and FT-IR ATR studies), and colloidal stability (via light scattering and electrokinetic potential measurements), blood compatibility (exploiting blood sedimentation experiments, smearing tests, WBC viability factor determination, and protein corona formation in blood serum) of the core–shell P(PEGMA-AA)@MNPs. In addition, we report on the excellent applicability of the P(PEGMA-AA)@MNPs for MRI contrast enhancement.

## 2. Materials and Methods

Magnetite nanoparticles (MNPs) were prepared by a co-precipitation method detailed in our previous and recent papers [32,33,34]. The crystalline structure of the synthesized iron oxide was identified as magnetite (Fe_3_O_4_) [35]. The average diameter of the MNPs was ~10.2 nm, determined from the broadening of the most intensive peak of the XRD pattern by using the Scherrer equation [34]. The primary core size of the synthesized MNPs was ~10 nm as determined by using a Philips CM-10 transmission electron microscope (Philips Electron Optics, Eindhoven, The Netherlands) with an accelerating voltage of 100 kV.

Acrylic acid (AA), polyacrylic acid homopolymer (PAA, *Mw* = 1800 g/mol), L-ascorbic acid, ethyl 2-bromoisobutyrate, 1,1,4,7,10,10-hexamethyltriethylenetetraamine (HMTETA), and trifluoroacetic acid were used as received. Poly(ethylene glycol) methyl ether methacrylate macromonomer (PEGMA300, *Mn* = 300 g/mol) was used after purification by passing through a column filled with neutral Al_2_O_3_. *tert*-Butyl acrylate (tBuA) was purified via vacuum distillation. Toluene was distilled over sodium/benzophenone and dichloromethane over calcium hydride. Cu(I)-chloride was stirred with acetic acid overnight, filtered, and washed with absolute ethanol and diethyl ether before use. All the small and macromolecular compounds used in this study were purchased from Sigma-Aldrich (Darmstadt, Germany)

The P(PEGMA) homopolymer and the P(PEGMA-AA) copolymer were synthesized via quasi-living atom transfer radical polymerization (ATRP), as detailed previously [31]. Toluene as solvent, ethyl 2-bromoisobutyrate as initiator, HMTETA as complexing agent, and Cu(I)-chloride as catalyst, were used. A starting random copolymer P(PEGMA-*co*-tBuA) was prepared using PEGMA300 and tBu-A monomers. The ATRP reaction mixtures were purified by passing through a neutral Al_2_O_3_ column. The P(PEGMA-*co*-tBuA) copolymer was transformed to the acrylic acid form via acidic hydrolysis, followed by stirring overnight and precipitating in hexane. The structure of the obtained comb-like copolymer product is shown in Table 1.

Number average molar masses of the obtained PPEGMA and P(PEGMA-*co*-tBuA) polymers were determined by gel permeation chromatography in tetrahydrofuran. We used the mass of the polymers or the molar amount of carboxyl groups to express the concentration of the different compounds.

Potentiometric acid−base titrations of P(PEGMA-AA) and AA were performed at 0.005, 0.05, and 0.5 M ionic strength of NaCl background electrolyte according to the procedure described previously [36]. The equilibrium method was used in the titrations with equilibrium criterion ΔpH/min < 0.01.

The adsorption of AA and P(PEGMA-AA) on magnetite was studied in batch adsorption experiments. In 10 mL total volume, 0.1 g (AA isotherms) or 0.01 g (P(PEGMA-AA) isotherms) of iron oxide was equilibrated with the surface modifying agents at concentrations from 0 to 2 mmol/g MNP at fixed pH and ionic strength (pH ~ 6.5 and *I* = 10 mM (NaCl)). The pH was adjusted to ~6.5 ± 0.2 using 0.01 and 0.1 mol/L NaOH or HCl solutions. After 1 day of standing, the pH of the samples was checked again. The solid phase was separated from the equilibrium supernatant by centrifugation (60 min, 14,000 rpm) assisted by the addition of concentrated NaCl solution and magnetic separation by means of a permanent NeFeB magnet (0.17 T). UV—vis spectrophotometry (USB4000 Fiber Optic Spectrometer, OceanOptics, Winter Park, FL, USA) was applied for determination of the equilibrium concentrations using the same method as for PAA [37]. The adsorbed amount (*n*^σ^) was expressed in the molar amount of carboxylic moieties (mmol COOH/g MNP), and calculated as *n*^σ^ = *V* (*c*_0,COOH_ − *c*_e,COOH_)/*m*, where *c*_0,COOH_ and *c*_e,COOH_ are the added initial and the equilibrium concentrations of the adsorbates, respectively, *V* is the total volume of the solution phase, and *m* is the mass of MNP. The experiments were repeated three times, and the results presented are the averages. The error was equal or less than ±0.1 for P(PEGMA-AA) and ±0.01 mmol/g for AA isotherms.

FT-IR ATR spectra were recorded with a Bio-Rad Digilab Division FTS-65A/896 spectrometer (with MCT detector) using a Harrick’s Meridian Split Pea Diamond ATR accessory (Bio-Rad Digilab Division, Cambridge, MA, USA). The absorbance of the samples was measured in single reflection mode over the 400−4000 cm^−1^ range (with resolution of 2 cm^−1^), accumulating 1024 scans. Magnetite dispersions (MNP, AA@MNP, P(PEGMA)@MNP and P(PEGMA-AA)@MNP) and the adsorbate solutions (AA, P(PEGMA) and P(PEGMA-AA)) were dripped and dried on the crystal surface. The pH of all samples was set to ~6.5, and the ionic strength was 10 mM. The monomer and polymer loadings of the coated MNP samples were 0.2 and 1 mmol/g, respectively. The background spectra were measured on clean and dry diamond crystal.

The zeta potential of the uncoated magnetite and the adsorbate-loaded nanomagnets (AA@MNP, P(PEGMA)@MNP and P(PEGMA-AA)@MNP) was determined in a Nano ZS (Malvern Instruments Ltd., Malvern, UK) dynamic light scattering (DLS) apparatus with a 4 mW He−Ne laser source (*λ* = 633 nm). The electrophoretic mobilities were recorded at 25 ± 0.1 °C using disposable zeta cells (DTS 1070, Malvern Instruments Ltd., Malvern, UK) and the Smoluchowski equation was applied to convert them to zeta potentials. The accuracy of the measurements is ±5 mV, and the zeta-standard of Malvern (−55 ± 5 mV) was used for calibration. The dispersions were diluted to give an optimal intensity of ∼10^5^ counts per second. Prior to the measurements, the samples were homogenized in an ultrasonic bath for 10 s, after which 2 min relaxation was allowed. The influence of adding AA and polymers (0−2 mmol/g) on the zeta potential of MNPs was determined at pH ~ 6.5 and *I* = 10 mM (NaCl). The pH-dependent surface charging properties of the naked and coated nanomagnets were studied from pH ~ 3 to ~10 at *I* = 10 mM.

The average particle size of bare magnetite and coated core–shell nanoparticles was determined at 25 ± 0.1 °C using a Nano ZS (Malvern Instruments Ltd., Malvern, UK) apparatus operating in backscattering mode at an angle of 173°. The solution conditions were the same as in the electrophoresis measurements: the added amounts of P(PEGMA-AA) varied between 0 and 2 mmol/g MNP, the pH range between ~3 and ~10, and the ionic strength (*I*) was 10 mM (NaCl). The aggregation state of the nanoparticles in the aqueous dispersions was characterized by the intensity average hydrodynamic diameter (*Z*_ave_) values. We used the second- or third-order cumulant fit of the autocorrelation functions, depending on the degree of polydispersity. The variation of *Z*_ave_ values was less than 5% for primary particles, and the error definition becomes irrelevant for large polydisperse aggregates.

Hemocompatibility of P(PEGMA-AA)@MNPs, i.e., their interaction with human blood, was studied in erythrocyte sedimentation rate (ESR) experiments utilizing a Sedi-15 automated sedimentation rate measuring device (BD Inc., Franklin Lakes, NJ, USA) and Seditainer 1.8 vacutainer tubes (BD Inc., Franklin Lakes, NJ, USA), as given in our previous publication [38]. P(PEGMA-AA)@MNP samples were mixed at room temperature with citrate-anticoagulated blood of three healthy donors to achieve a concentration of 0.24 mg/mL. The experimental error given by the producer is ±3 mm/h. Three replicates of the ESR measurements were performed for all donors. Peripheral blood smear tests of the whole blood (EDTA-anticoagulated) of the donors were carried out by an automated slide preparation system (Sysmex SP4000i, Sysmex, Kobe, Japan) at room temperature using the May-Grünwald Giemsa (MGG, Biolyon, Dardilly, France) staining technique in a CellaVisionTM DM96 automation device (CellaVision AB, Ideon, Science Park, Lund, Sweden). The influence of P(PEGMA-AA)@MNPs on platelet aggregation was studied at a concentration of 0.4 mg/mL. The acquisition and classification software of the instrument was used to differentiate between normal and abnormal cells (white blood cells (WBCs), red blood cells (RBCs), and platelets (PLT)).

The WBC viability factor (WVF), i.e., the fraction of viable white blood cells was determined by using a CELL-DYN Sapphire hematology analyzer (Abbott Diagnostics, Santa Clara, CA, USA). This routine diagnostic method differentiates between necrotic, apoptotic, and normal cells in anticoagulated blood via selective staining of nuclei with a cell membrane-impermeable fluorescent dye, propidium iodide. After hydrodynamic focusing, WVF was determined by the emitted red fluorescence (at 617 nm) of the dye bound to the nucleic acid of injured or dead cells. The effect of P(PEGMA-AA) coated nanoparticles on WBC viability was examined at 37 °C. The P(PEGMA-AA)@MNP concentration in blood was 0.4 mg/mL. The WVF values were determined 10, 30, 60, 90, 140, and 240 min after mixing the MNPs with blood at a concentration of 0.4 mg/mL. The accuracy of the measured WVFs was above 97%, as calculated from 5 parallel runs. In addition to white blood cells, the viability of other cellular elements, such as red blood cells (RBCs), neutrophils, lymphocytes, and monocytes, was determined as well. Impedance measurements were used to determine the cell counts of WBC, RBC, and PLT (all viable, damaged, and dead cells) and their volumes as well.

For testing the MNP interactions with human plasma (HP), the blood of six healthy donors (age of 30 to 64, five females and one male) was collected in the Institute of Laboratory Medicine of the University of Szeged, according to the routine blood drawing practice of the Institute. HP was separated from the blood in EDTA-anticoagulated tubes (Seditainer 1.8 vacutainer tubes, BD Inc., USA), using five tubes for each donor, and then centrifuged. The plasma from all samples was pooled, and a second step of centrifugation at 16,000 rpm for 10 min was applied to remove residual cells and cell debris. The total protein content was measured in the pooled sample as 65 g/L. The within-subject and between-subject biological variation in total protein content was 2.75 and 4.7, respectively, based on the Westgard database [39]. A plasma pool was used to get identical protein content in all experiments. After freeze-drying the product in a Flexi-Dry μP lyophilizer (FTS Systems) using liquid nitrogen, it was stored at −18 °C in small aliquots, and separately thawed at room temperature for individual experiments. For protein corona formation experiments in HP, the thawed aliquots were dissolved in ultrapure (UP) water to make up the original 100% concentration. The stock solution of HP was further diluted by using Tris-buffered saline solution from Sigma-Aldrich, Darmstadt, Germany (pH ~ 7.4, I ~ 154 mM). The experiments with the bare and P(PEGMA-AA)-coated MNPs were performed in three replicates using separate plasma aliquots. Freeze-dried plasma was preferred for the series of our room temperature experiments, because fresh plasma can only be stored for a maximum of three days.

The evolution of the average particle size of P(PEGMA-AA)@MNPs in the course of protein corona formation in human plasma was studied at 25 ± 0.1 °C, both as a function of HP concentration and time. The HP concentration was increased from 0 to 80 *v*/*v* %, and the measurements were done at 3 min and at 20 h after MNP incubation in plasma. For particle size determination, the same apparatus was used as above, and the experimental conditions, data processing, and accuracy of measurements were also the same. The pH and ionic strength were fixed by using the Tris-buffered saline medium. The changes in the zeta potential due to protein corona formation were determined in electrophoretic mobility measurements at 25 ± 0.1 °C by using the same apparatus, experimental conditions, and evaluation as above. The experiments were performed at 20 h after MNP incubation in plasma.

The MRI contrast enhancement efficiency of the P(PEGMA-AA)@MNP was studied by using a clinical MRI instrument GE Excite HDxt (GE Medical Systems, Milwaukee, WI, USA) with a standard “birdcage” head coil at magnetic field strength of 1.5 T. The MNP dispersions were prepared at nominal Fe concentrations of 0.009, 0.018, 0.036, 0.072, 0,107, 0.143, and 0.179 mM. The exact iron content of the samples was measured by inductively coupled plasma optical emission spectroscopy method using an Optima 7000 DV ICP-OES instrument (Perkin-Elmer, Shelton, CT, USA). Samples (4 mL) were placed in a plastic box filled with water, and put in the center of the head coil. The MR images were acquired at echo delay times (TE) of 10, 20, 30, 40, 60, 120, 180, and 240 ms by applying a radio frequency repetition time (TR) of 3000 ms. The *r*2 relaxivity, quantifying the contrast enhancement efficiency of the P(PEGMA-AA)@MNPs, was determined as the slope of the 1/*T*2 vs. Fe concentration plot. The quality of fitting was estimated by the coefficient of determination (*R*-squared).

## 3. Results and Discussion

Our novel brush copolymer P(PEGMA-AA) (Table 1) had been designed to combine such structural elements that assist its durable stealth layer formation on the MNPs which, in turn, ensures colloidal stability, blood compatibility, and sufficient circulation times for biomedical applications. The AA units (acrylic acid units), in general, bind to the positively charged MNP surface (the net surface charge is positive at pH ~ 6.5 applied in the binding process) both in electrostatic and surface complexation mechanisms [37]. This binding is further strengthened by the presence of hydrophobic –CH_3_ groups of the backbone of P(P(EGMA-AA) [31]. In addition, the AA anionic moieties and PEG chains together provide a strong electrosteric (i.e., combined electrostatic and steric) repulsion between the P(PEGMA-AA)@MNPs, leading to excellent colloidal stability. For deeper insight into the process of binding, the results discussed here will be compared with those obtained for the basic molecules of P(PEGMA-AA) components, i.e., small molecular acrylic acid (AA), comb-like homopolymer P(PEGMA), and polyacrylic acid homopolymer (PAA). The characteristics of the examined molecules are collected in Table 1. We further anticipated that the pending PEG chains can hinder the anionic carboxylates from direct exposition to biomacromolecules, allowing for long circulation times (beneficial in MRI applications) due to the prevention of nonspecific protein adsorption, protein denaturation, and phagocytic clearance of the particles.

### 3.1. pH- and Ionic Strength-Dependent Dissociation of P(PEGMA-AA)

**Figure 1:**
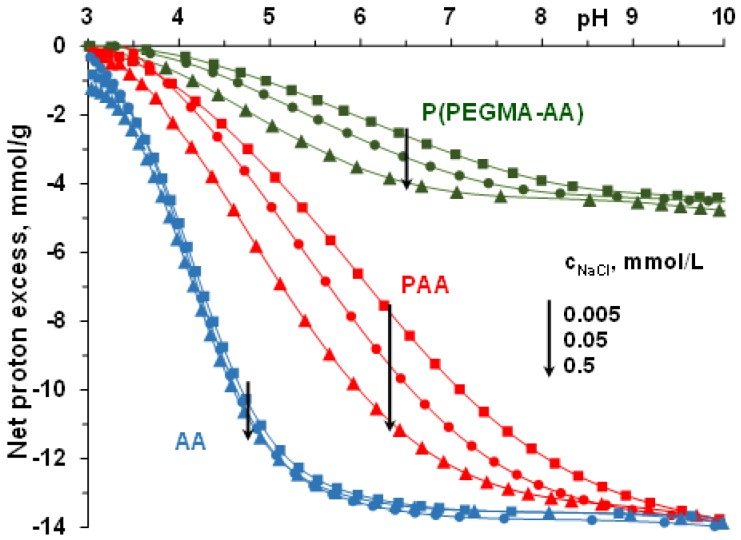
pH- and ionic strength-dependent dissociation of acrylic acid (AA), polyacrylic acid (PAA) and P(PEGMA-AA): –COOH + OH^−^ → –COO^−^ + H_2_O reaction taking place with base addition. The lines connect experimental data points.

The binding of polycarboxylates to MNP surfaces is strongly influenced by the density and degree of dissociation of acidic groups, and so, it is of primary importance to determine the degree of dissociation of P(PEGMA-AA) at the pH and ionic strength conditions applied in the process of binding on MNPs. The dissociation of carboxylic groups of the AA segments in P(PEGMA-AA) was measured in potentiometric acid–base titrations in comparison with that of the same groups in AA monomers and PAA homopolymers (Figure 1). See more about the acid–base titration method in the point “Potentiometric Acid–Base Titration of Polyelectrolytes” in Supplementary Material. The dissociation of carboxylic moieties reaches limiting values characteristic of the specific compounds. The nominal value of the molar amount of carboxylic groups is 14 mmol/g in both AA and PAA (Table 1), and their experimental amount from titrations is ~13.9 mmol/g (see the limiting values of net proton consumption at pH ~ 10). The titrated specific amount of dissociable carboxyl groups of P(PEGMA-AA) is 4.6 mmol/g, somewhat smaller, but very close to the theoretic value 5 mmol/g, calculated from PEGMA/AA monomer ratio of 1:2.33 in ATRP synthesis (Table 1). The experimental amounts of carboxylic groups in AA and P(PEGMA-AA) were used for further concentration calculations in all the experiments.

The net proton excess vs. pH curve of AA is independent of ionic strength, as is expected for small molecules. The characteristic ionic strength dependence of PAA dissociation reveals the formation of an electric double layer around the polyanion, screened increasingly by increasing the ionic strength of the indifferent background electrolyte [40]. A very similar ionic strength dependence of the net proton excess vs. pH curves was found for the P(PEGMA-AA) copolymer. The latter similarity is somewhat surprising in the light of the different charge distribution of the two polyelectrolytes: while every monomeric AA unit of PAA is a charge carrier, in P(PEGMA-AA), uncharged PEGMA units separate the AA charges. The similar polyelectrolyte feature of PAA and P(PEGMA-AA) may be explained by the presence of –CH_3_ groups in the backbone of the latter. The hydrophobic methyl groups can lead to more contracted conformation in water relative to that of the fully hydrophilic PAA, resulting in a similar volume charge density of the two polyelectrolytes.

### 3.2. PEGylation of MNPs by Using P(PEGMA-AA)

**Figure 2:**
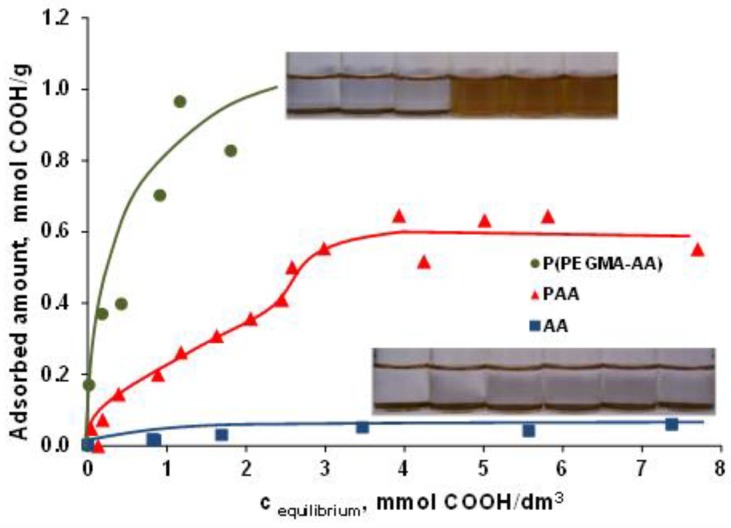
The adsorption isotherm of P(PEGMA-AA) in comparison to AA, PAA [37] isotherms on magnetite surface at pH ~ 6.5 and 10 mM NaCl. The equilibrium concentration and adsorbed amount are related to the molar amount of carboxylic groups (COOH). The error bars are omitted for clarity, and lines are drawn to guide the eyes. The pictures of the adsorption series are taken at increasing equilibrium concentrations of P(PEGMA-AA), from 0 to 2.06 mmol/dm^3^, and of AA from 0 to 7.4 mmol/dm^3^.

For binding of P(PEGMA-AA) on the MNP surface, we exploited the self-organizing property of polyelectrolytes at solid–liquid interfaces. Adsorption isotherms were measured to determine the optimal amount of polyelectrolyte necessary to coat the entire NP surface. The P(PEGMA-AA) and AA adsorption isotherms were measured on the magnetite nanoparticle surface at pH ~ 6.5 and *I* = 10 mM and are shown in Figure 2, in comparison with the previously published isotherm of PAA [37] adsorption under same conditions. P(PEGMA-AA) adsorbs on magnetite with high affinity. At low added amounts of P(PEGMA-AA), almost all molecules become bonded to the surface. The isotherm differs strongly from that of PAA and AA. AA is able to adsorb in an amount corresponding to the positive surface charge of MNPs at pH = 6.5 and *I* = 10 mM (0.06 mmol COO^−^ (AA)/g MNP and 0.05 mmol ≡Fe−OH_2_^+^/g MNP [37], respectively), suggesting that the adsorption is driven by electrostatic interaction and stops at charge compensation. PAA, similarly to polyelectrolytes in general, reaches adsorbed amounts, greatly overcompensating for the original positive surface charge of MNP (0.05 mmol/g at pH ~ 6.5 and 10 mM NaCl). The isotherm is not of a high-affinity type, and the saturation level is about half of that of P(PEGMA-AA). The P(PEGMA-AA) isotherm is more similar to that of PAM (poly(acrylic acid-*co*-maleic acid [38], not shown here) than PAA or AA. The presence of PEGMA segments appears to have a definite impact on the adsorption mechanism. PAM adsorption was found to proceed via direct iron complexation of the bicarboxylic maleic acid monomer and H-bonding of non-dissociated carboxyls, while PAA adsorbed solely via H-bonding of non-dissociated carboxylic moieties. The presence of CH_3_ groups on the backbone and at the end of each PEG chains of P(PEGMA-AA) provide an amphiphilic character to the molecule, most likely leading to a highly preferred orientation and accumulation of the comb-like polymers at the surface of MNPs. This self-assembly is greatly assisted by the electrostatic attraction of carboxylate moieties. The entropy gain here, similarly to that in the formation of micelles of amphiphilic molecules, is responsible for self-assembling of P(PEGMA-AA) at the MNP/aqueous solution interface. The driving force is known to be the loss of direct contact between the hydrophobic moieties (CH_3_ groups here) and water molecules [41]. The latter process is called hydrophobic hydration, and can be the reason for the high-affinity feature of the adsorption isotherm of P(PEGMA-AA).

As it was proven in transmission electron microscopy (TEM) experiments (see our previous publication [31]), P(PEGMA-AA) formed an intact polymer layer around the particles upon binding. TEM analysis gave a value for coating layer thickness of 2–2.3 nm. For brush-like PEG chains of 4.5 ethylene glycol units (in P(PEGMA-AA)) the expected coating thickness would be around 1–2 nm, and an additional layer thickness can be expected from the polymer backbone.

### 3.3. FT-IR ATR Study of the Binding Chemistry of P(PEGMA-AA) on MNPs

**Figure 3:**
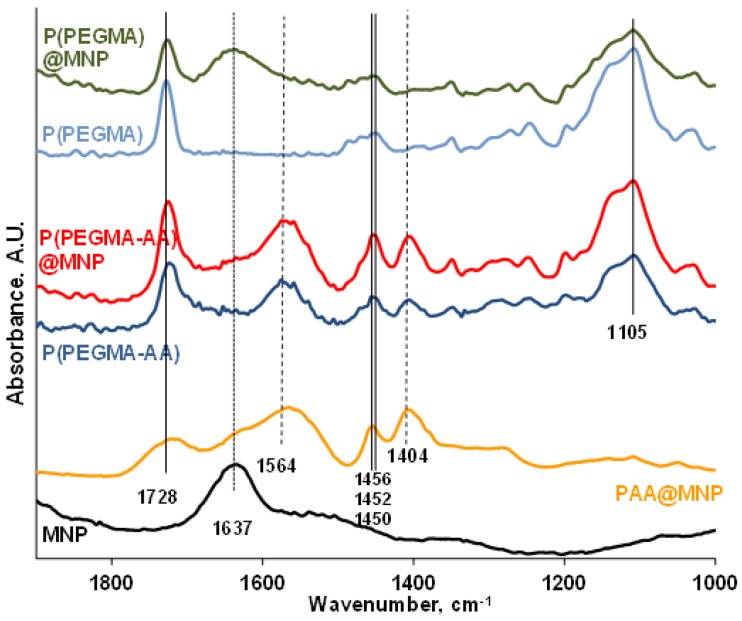
FT-IR absorption spectra of P(PEGMA-AA) and P(PEGMA) before and after adsorption on MNP in the range of 1000–1900 cm^−1^. The spectrum of PAA@MNP [37] are also plotted for comparison. The samples were dried from pH ~ 6.5 and *I* = 10 mM medium on ATR diamond crystal.

FT-IR ATR spectroscopy was used to study the molecular interactions between P(PEGMA-AA) and the MNP surface. The spectra in Figure 3 show absorption peaks in the 1900–1000 cm^−1^ wavelength range, most characteristic for oxygen-containing organic compounds.

The spectra of P(PEGMA) and PAA are useful for identification of separate peaks in P(PEGMA-AA). The carbonyl band of the ester groups of P(PEGMA) is at 1728 cm^−1^, while that of the carboxylic groups of PAA is at 1699 cm^−1^. In the P(PEGMA-AA) molecule, there is a single carbonyl frequency at 1723 cm^−1^, resembling a combination of the carbonyls of PEGMA ester and dissociated (acrylate) AA, and suggesting an interaction between those via, for example, H-bonding [42]. The carbonyl C=O stretching of P(PEGMA) also shifted from 1728 to 1726 cm^−1^ upon adsorption on MNP (compare the P(PEGMA) and P(PEGMA)@MNP spectra), indicating the probability of a similar H-bonding with ≡Fe–OH surface hydroxyl groups, such as with AA carboxyls of P(PEGMA-AA). It is interesting, however, that the vibrational energy of the H-bonded C=O groups of P(PEGMA-AA) does not change upon adsorption on MNPs. This indicates that either the carbonyls do not participate in the adsorption interaction, or that they form new H-bonds with the ≡Fe–OH surface hydroxyls on account of the original H-bonds between C=O (PEGMA) and –COOH (AA). Other characteristic peaks at 1564 cm^−1^ (–COO^−^ asymmetric vibrations of PAA and P(PEGMA-AA)); 1456, 1452, and 1450 cm^−1^ (CH_2_/CH_3_ C–H bending of PAA, P(PEGMA-AA), and P(PEGMA), respectively); 1404 cm^−1^ (–COO^−^ symmetric vibrations of PAA and P(PEGMA-AA)) and 1105 cm^−1^ (C–O–C ether stretching bands of P(PEGMA), clearly did not shift upon polymer adsorption at all. The inertness of the acrylic carboxylate groups of P(PEGMA-AA) in the adsorption on MNPs is in contrast with the behavior of the carboxylates of small molecular AA (see in Figure S1, and discussion in Supplementary Material). The Fe–OH vibration of magnetite at 1637 cm^−1^ disappears from the P(PEGMA-AA)@MNP spectrum supporting the reliability of the H-bonding interaction of ≡Fe–OH (and/or ≡Fe–OH_2_^+^) with the ester and/or carboxyl carbonyls of P(PEGMA-AA) and its transition to Fe–O–C–R bonds. Accumulating near the MNP surface, the original carbonyl–carboxylate H-bonds of P(PEGMA-AA) can possibly lose the structured water molecules due to simultaneous crowding of hydrophobic CH_3_ moieties in the adsorbed layer (see in section “PEGylation of MNPs by Using P(PEGMA-AA)”). The loss of the stabilizing structured water can lead to H-bond decomposition and formation of lower energy surface Fe–O–C–R bonds with further water release.

The spectra in the ranges of 4000–2400 and 800–500 cm^−1^ are discussed in Supplementary material (Figure S2).

### 3.4. Evolution of the Colloidal Stability of Core–Shell MNPs

**Figure 4:**
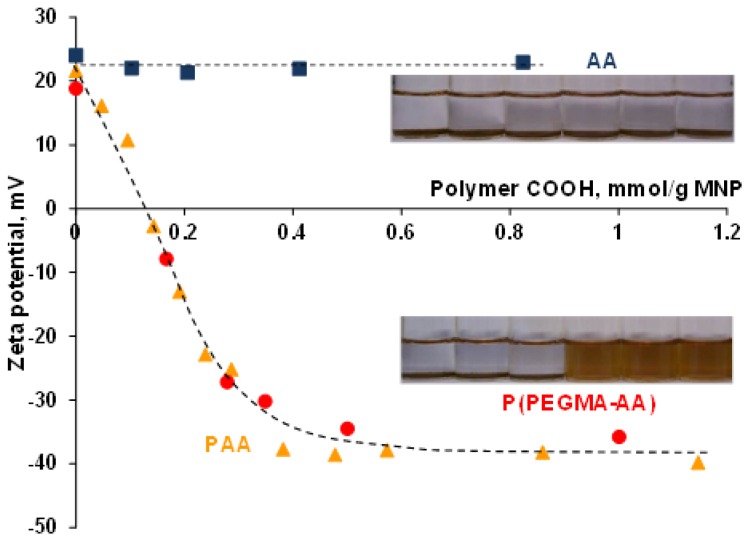
Zeta potentials of coated nanomagnets as a function of P(PEGMA-AA) (red), PAA (orange) [37], and AA (blue) loadings, calculated on the basis of molar amount of carboxylic groups (0–1.2 mmol/g). The measurements are done at pH ~ 6.5 and *I* = 10 mM (NaCl).

In electrokinetic potential measurements, we found that, despite the differences in the characteristics of binding, the charge of P(PEGMA-AA)- and PAA-coated nanoparticles is the same if the amounts of bound AA groups (mmol COOH/g MNP) are equal (Figure 4). The charge of bare magnetite nanoparticles in aqueous dispersions is positive at the pH and ionic strength of our adsorption experiments (pH = 6.5 and *I* = 10 mM). The net amount of ≡Fe−OH_2_^+^ surface groups, i.e., their excess over ≡Fe−O^−^ groups, is ~0.05 mmol/g [38]. Binding of anionic adsorbates on the MNP surface alters the electrokinetic potential by compensating (or overcompensating) the positive original surface charge of the MNPs. The changes in the zeta potential to P(PEGMA-AA) adsorption are shown in Figure 4, in comparison with that of PAA and AA. 

The pictures inserted in Figure 4 show the changes in the aggregation state of MNPs in the course of coating agent addition. The aggregation behavior was similar for AA and P(PEGMA)-coated and for P(PEGMA-AA)- and PAA-coated MNPs (upper and lower pictures, respectively). Particle aggregation was observed between ~+30 and ~−30 mV of zeta potential in the absence of sufficient electrostatic repulsion between particles. The amount of carboxylic moieties to achieve the maximal value of (negative) zeta potential is ~0.5 mmol/g MNP for both polyelectrolytes.

On the other hand, if the zeta potential evolution was represented in function of coating agent weight (g polymer/g MNP), as it is accustomed in literature, P(PEGMA-AA) would appear to be less efficient in electrostatic stabilization of MNPs (see Figure S3). Although this type of evaluation has practical advantages, it can lead to false conclusions about the capabilities of different polyelectrolytes in stealth layer formation. More detailed discussion of the zeta potential changes due to P(PEGMA-AA) binding is given in Supplementary Material (“Additional discussion on the changes in zeta potential due to P(PEGMA-AA) addition”).

**Figure 5:**
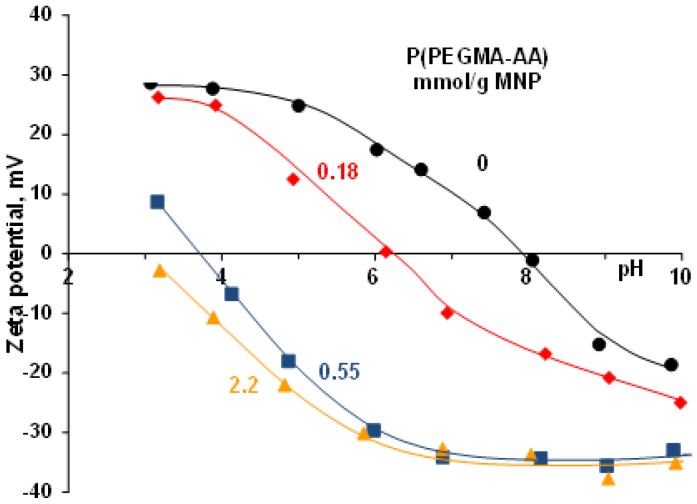
The pH-dependent zeta potentials of P(PEGMA-AA)-coated nanomagnets at 0, 0.18, 0.55, and 2.2 mmol COOH/g MNP loadings at 10 mM NaCl. The error bars are omitted for clarity and lines are drawn to guide the eyes.

The pH dependence of the electrokinetic potential and hydrodynamic diameter of P(PEGMA-AA)@MNPs, and their aggregates at different P(PEGMA-AA) loadings, was studied in order to find the optimum loading to stabilize the MNPs in the widest possible pH range. Sufficient pH resistance is necessary for a multitude of biomedical applications. As is seen in Figure 5, the addition of increasing amounts of P(PEGMA-AA) to MNPs shifts the pH of the isoelectric point gradually to lower values, and widens the pH range of electrostatic stabilization.

At low pHs (below ~6, not significantly lower than the pH in biological environment), however, the electrokinetic potential of core–shell MNPs cannot be reduced below ~−30 mV (the electrostatic stability threshold) even at the highest P(PEGMA-AA) loading of 2.2 mmol/g. This alone would suggest a narrower pH range of colloidal stability of P(PEGMA-AA)@MNPs, as compared with that of PAA [37]- and PAM [38]-coated MNPs. However, DLS experiments proved the presence of well-stabilized primary particles at pHs 5 and 4, and 0.55 or 2.2 mmol/g polyelectrolyte loadings, in spite of the low absolute values of zeta potential (between ~−25 and ~−15 mV) (see Figure S4). As the electrostatic component to colloidal stabilization is exactly the same for the two kinds of core–shell MNPs (PAA and P(PEGMA-AA)-coated particles), the above result implies that the steric component, due to the presence of PEG chains in the stealth layer, has a determining role in particle stabilization. In addition, P(PEGMA-AA) pushed the acidic limit of the pH threshold of colloidal stability by 1 unit lower, relative to that of a similar comb-like polyelectrolyte PEG-acrylate-*co*-acrylic acid, (P(PEGA-AA), [32]). The only difference in the structures of the two PEG copolymers is the absence or presence of –CH_3_ groups in the backbone. Thus, it can be supposed that the better stabilization capability of P(PEGMA-AA) can be due to the improvement in self-organization on the surface of MNPs. Apparently, the presence of the hydrophobic –CH_3_ in the polyelectrolyte backbone prompts the formation of tighter and sterically more repulsive stealth layer.

### 3.5. Salt Tolerance of P(PEGMA-AA)@MNPs

Detailed analysis of the pH dependence of hydrodynamic size of P(PEGMA-AA)@MNPs at and below physiological salt concentrations [31] revealed that the polymer coating increased the average hydrodynamic diameter of naked MNPs by ~30 nm (from ~80 to ~110 nm), leaving the PDI (polydispersity index as given in Malvern software) values practically unchanged (i.e., 0.15 and 0.16, respectively). The large values of hydrodynamic diameters of well-stabilized naked and coated MNPs (as compared to their physical diameter from TEM, ~10 nm) are due to the formation of nanoparticle clusters, and this observation was also supported by the magnetic hyperthermia results.

**Figure 6:**
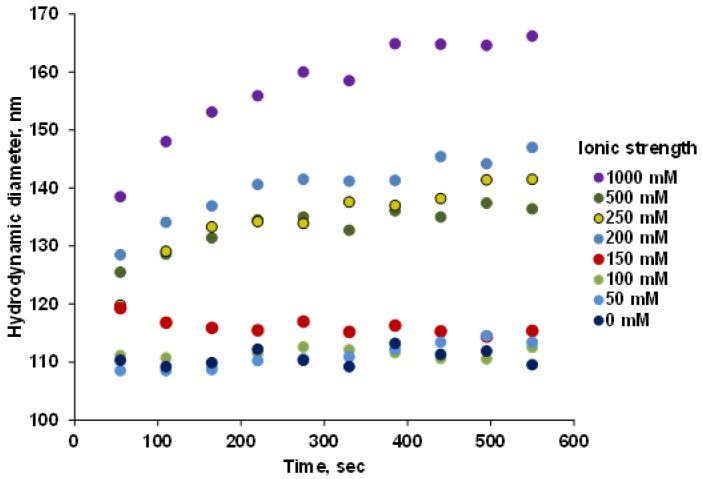
Changes in the kinetics of coagulation of P(PEGMA-AA)@MNP with 1.2 mmol COOH/g MNP loading measured at pH ~ 6.5 as a function of ionic strength.

The P(PEGMA-AA)@MNP dispersions were colloidally stable at 10 mM ionic strength when pH > ~4, and polyelectrolyte loadings above 0.55 mmol COOH/g MNP (Figure 5). The salt tolerance of the particles was tested in coagulation kinetics experiments at pH = 6.5 and 1.2 mmol/g P(PEGMA-AA) loading. Figure 6 shows that particle coagulation principally was not observed until raising the salt concentration to 150 mM.

At 150 mM NaCl, the ionic strength of blood, instant aggregation is seen, and the hydrodynamic diameter gradually decreases with time. At higher salt concentrations, 200, 250, and 500 mM, instant aggregation can also be seen, which is followed by moderate size increase over the span of experimental observation. Both below and above 150 mM NaCl, the feature of the kinetics of coagulation seems to be changing non-systematically with ionic strength or, rather, independently of that. The highly non-ideal aggregation behavior of the P(PEGMA-AA)-coated MNPs can be caused by steric adaptation of the polyelectrolyte shell to the increase in ionic strength. With increasing ionic strength, the repulsive potential of the particles decreases. This, in turn, promotes aggregation, which is counteracted by the dehydration effect of the salt. Dehydration reduces the thickness of the polyelectrolyte shell and the distance of closest approach and, so, the potential at this plane (somewhat proportional to zeta potential) increases, counteracting the charge-induced zeta potential decrease. In addition, the formation of a denser coating layer with strongly hydrophilic PEG units contributes to stabilization via steric effects.

The P(P(EGMA-AA)@MNPs show principally different salt tolerance behavior, as compared to all other carboxylated MNPs synthesized in our laboratory (e.g., PAA@MNP [37], PAM@MNP [38]), which is due to the efficient screening of acrylate charges by the ordered layer of PEG brushes. The inaccessibility of the charged moieties possibly prevents ion pair formation with background electrolyte cations and, thereby, the destabilization effect of the electrolyte is reduced. Thus, the core–shell MNPs behave similarly as uncharged hydrophilic particles or molecules possessing low pH- and salt sensitivity. Measured at 500 mM salt concentration, all the other core–shell MNPs aggregated in coagulation kinetics experiments to reach a hydrodynamic size of 400–600 nm at 600 s of measuring time, while the size of P(PEGMA-AA) MPNs did not exceed 140 nm. Even an extremely high salt concentration, 1000 mM, could not double the particle size, i.e., it increased from ~100 to ~170 nm.

### 3.6. Hemocompatibility of P(PEGMA-AA)@MNPs

**Figure 7:**
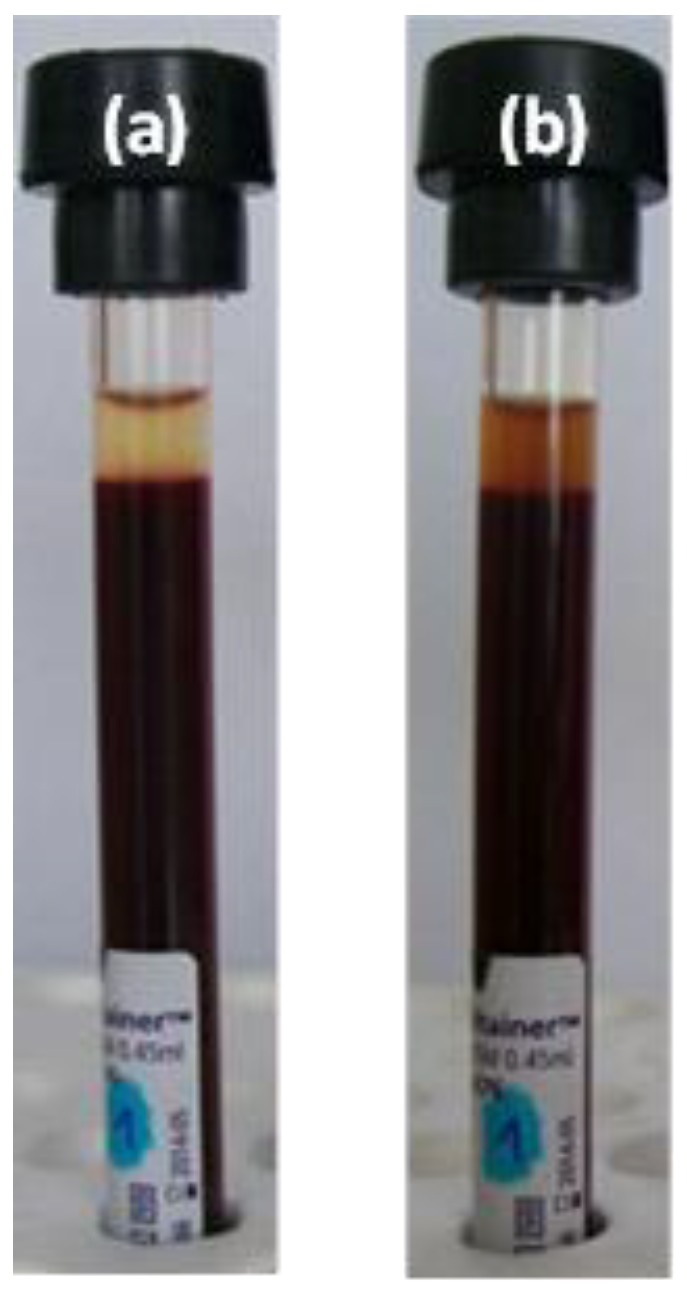
Sedimentation test results of sample from Donor #1: (**a**) vial contains original blood sample and (**b**) vial the sample with P(PEGMA-AA)@MNP added at 0.24 mg/mL concentration.

We demonstrated previously in biocompatibility experiments [31] that P(PEGMA-AA)@MNPs do not have any harmful effect on cell cultures of human origin. Since MRI contrast agents are injected intravenously, the P(PEGMA-AA)@MNPs should obey excellent blood compatibility as well, which we tested on whole blood samples. The blood sedimentation rate (ESR) analysis result of Donor #1 is seen in Figure 7. The rates of sedimentation are essentially identical, within the experimental error for both control blood sample and the sample containing the P(PEGMA-AA)@MNPs, i.e., 9 ± 2 and 5 ± 2 mm/h, respectively. Our previous studies of the effect of MNP addition showed that the same type of MNP can alter the ESR values by between −1 to +5 mm/h depending on the donor, while remaining in the normal range, below 22 mm/h. One example of this donor-dependent variability (−1, +5, and +1, for 3 donors) has been published by us earlier [38], the explanation of which requires additional studies.

The brown colour of the plasma in the right vial reveals the presence of MNPs. The cell viability experiments (see red blood cell count results below) may be used to exclude the contribution of hemolysis to plasma discoloration, as the number of whole red blood cells remained essentially constant during the 240 min of our experiment).

**Figure 8:**
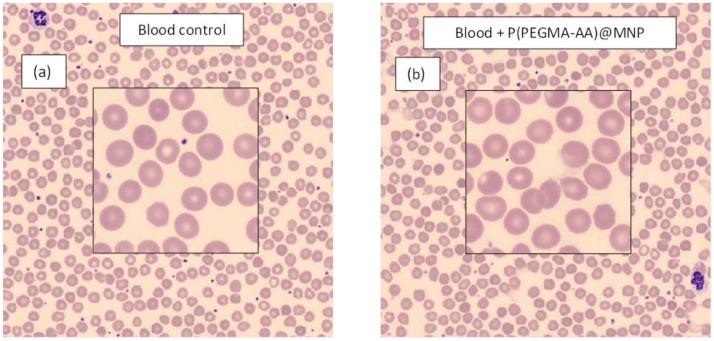
Whole blood smears from the sample of Donor #1 without (**a**) and with (**b**) P(PEGMA-AA)@MNP added at 0.4 mg/mL concentration. The plasma of the two samples are seen in the insets. Small dots in the magnified regions are thrombocytes. The nanoparticles are invisible at the magnification of the optical microscope.

The smears of control and MNP containing blood also did not reveal signs of coagulation (Figure 8). Nanoparticles are not seen at the resolution of the image of smear tests unless coagulation occurs. The small dots in the enlarged fields are non-aggregated thrombocytes. In our previous studies, we found strong aggregation in citrate-anticoagulated blood with well-stabilized polygallic acid- and PAA-coated MNPs [43] presumably due to iron dissolution by citric acid that could displace part of the polyelectrolytes in the MNP coating layer. Additional examples of thrombocyte aggregation induced by poorly stabilized MNPs are given in [44]. For this reason, we use EDTA-anticoagulated blood in the smears.

**Figure 9:**
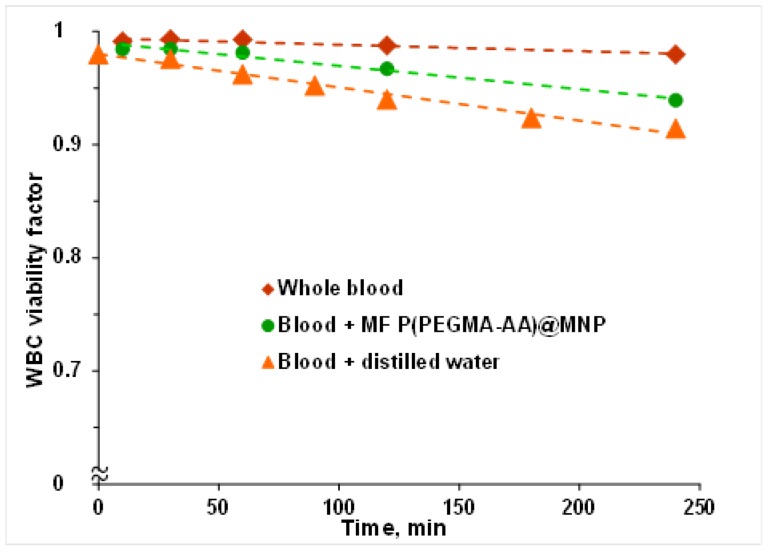
Changes in white blood cell viability factor as a function of time in whole blood and blood diluted with magnetic fluid (the P(PEGMA)@MNP concentration in blood is 0.4 mg/mL) and distilled water.

The results of WBC viability factor (WVF) measurements are presented in Figure 9. Comparing the WVF results for whole blood and blood samples diluted with distilled water and with P(PEGMA-AA)@MNP magnetic fluid in the same volume ratio, the WV factor decreased most intensely due to the addition of distilled water. This shows that the viability decrease is caused primarily by osmotic effects. The magnetic fluid has a smaller influence because it contains the core–shell MNPs and, consequently, the volume fraction of water (responsible for the osmotic action) is less than 1. Despite the measurable changes in WVF, the effect of the magnetic fluid is negligibly small, leading to less than 5% decrease during the four hours of experiment. Cell counts (cells/L) varied at 5.45–6.40 × 10^6^ (WBC), 3.13–3.76 × 10^9^ (RBC), and 1.74–2.09 × 10^6^ (PLT), and the variation of cell volumes was less than 5% in the presence of MNPs. No trends were observed for cell count and volume variations as a function of the concentration of MNPs in the blood. The measured results for white cell subpopulations (neutrophils, lymphocytes, and monocytes) also show minor impact of MNP addition on the recognition system. The percentage viability values of neutrophils (pNEU) changed in the course of the experiments, from 57.36 to 57.42 (in reference sample without MNP from 55.8 to 54.52), that of lymphocytes (pLYM) from 32.03 to 33.19 (reference 33.79 to 33.96), and of monocytes (pMON) from 7.88 to 6.45 (reference 7.05 to 8.35). Comparing these values with the reproducibility requirement given by producer (pNEU: ±8.55%, pLYM: ±5.10%, pMON: ±8.90%), we have to conclude that the change of pNEU and pLYM values in time is not even higher than their reproducibility limits. We observed a higher change only for pMON, however, this was the case for both the MNP containing and reference samples. The latter also reflects the absence of sensitivity of blood to the presence of P(PEGMA-AA)-coated nanoparticles.

For MRI applications, there should be no interaction between the contrast agent nanoparticles and blood plasma proteins, or at least, nonspecific interactions must be absent [45]. Instead of concealing, non-specific attachment of proteins help nanoparticle recognition and clearance from circulation. The interaction between P(PEGMA-AA)@MNPs with plasma proteins were tested in DLS experiments and electrokinetic measurements.

Changes in the hydrodynamic diameter of the MNPs were measured upon contact with human plasma of increasing concentrations. The measurements can reflect simultaneously both aggregation and changes in the coating layer thickness.

**Figure 10:**
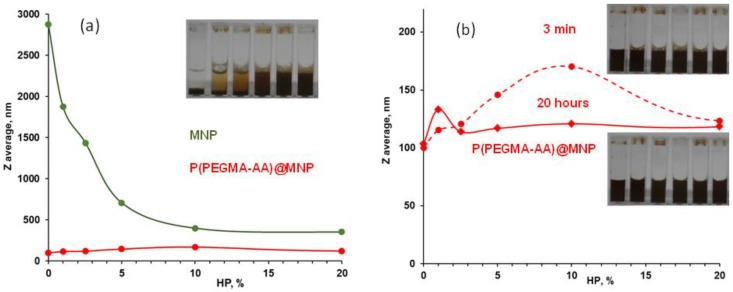
Changes in the hydrodynamic diameter of naked MNP (green) and P(PEGMA-AA)@MNP (red) as a function of the concentration of HP measured 3 min (**a**) and P(PEGMA-AA)@MNPs at 3 min and 20 h after dispersing in HP solution (**b**).

The hydrodynamic diameter of naked and P(PEGMA-AA)-coated MNPs (Figure 10) was higher at low plasma concentrations, and lower at high plasma concentrations. The low HP concentration regime resembles the conditions at the locus of intravenous administration in that the ratio of proteins to nanoparticles is very low. Adsorption of only a few protein molecules reduces considerably the size of naked MNP aggregates and somewhat enhances that of P(PEGMA-AA)@MNPs. In both cases, this is a likely sign of the initiation of protein corona formation. The size of naked MNPs remains above 400 nm at all HP concentrations, meaning that the particles of ~10 nm diameter are dispersed by protein adsorption, but the corona is formed around aggregates of naked MNP. By contrast, the size of P(PEGMA-AA)-coated MNPs increased less than twofold, revealing that, instead of particle aggregation, the formation of a protein corona is more likely. At higher HP concentrations (up to 80% of the original HP solution) aggregates do not form, but the size of protein corona increases gradually, as seen from the increase in *Z*_ave_ from ~120 nm at 20% to ~180 nm at 80% HP (see Figure S5). The polydispersity index was between 0.6 and 1 for the aggregated naked MNPs (in the whole HP concentration range) and between 0.2 and 0.4 for P(PEGMA-AA) MNPs, which also supports the lack of aggregation in the latter systems. The aggregation state of the dispersions was visualized in sedimentation experiments as seen in the pictures. Indeed, no sedimentation was observed in dispersions of P(PEGMA-AA)@MNP in HP solutions. The initially higher hydrodynamic diameter values (measured at 3 min after mixing the MNP dispersions with HP solutions) decreased with time, which is probably the sign of formation of denser protein corona with time. For the naked MNPs, the size decrease with time is about 20% [46]. This is likely due to some further decrease in the size of aggregates. On the other hand, for P(PEGMA-AA)@MNPs, a consistent size change with time cannot be observed. After the initial oscillations with HP concentration, the hydrodynamic diameter steadily increases at higher HP concentrations, most likely due to the dynamic character of the protein corona (see Figure S5).

**Figure 11:**
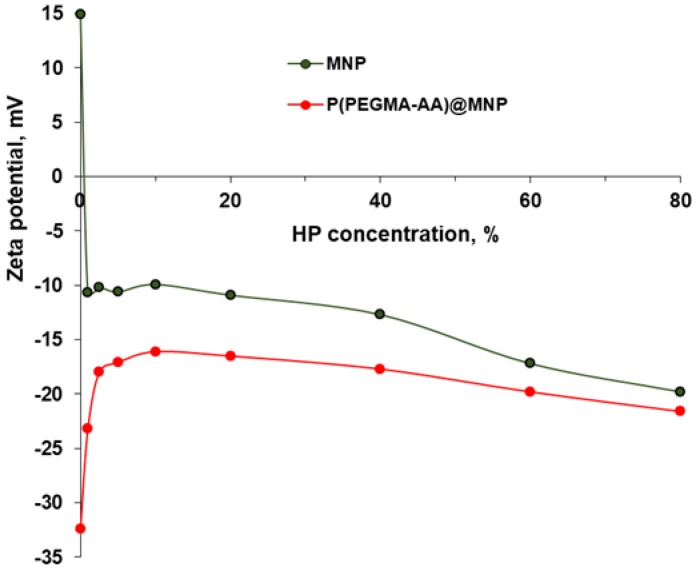
Changes in the electrokinetic potential values of naked and P(PEGMA-AA)-coated MNPs in the course of protein corona formation in HP solutions prepared with Tris-buffered saline solution, as measured at 20 h after the MNP-HP dispersion preparation.

The zeta potential changes in the course of protein corona formation (Figure 11) reflect the protein corona formation both around naked MNP aggregates and well-dispersed P(PEGMA-AA)@MNP particles. The aggregates of originally positively charged naked MNPs (pH ~ 6.5 and *I* = 10 mM) get overcharged negatively, and the negative zeta potential increases with increasing HP concentration. As the zeta potential of HP solutions is ~−5mV, the much higher negative potentials measured for both naked and P(PEGMA-AA)-coated MNPs indicate dense packing of HP in the protein corona. The adsorption of HP on P(PEGMA-AA)@MNPs decreases their originally high negative zeta potential (−32 to −16 mV), probably due to shifting of the plane of shear by the protein corona farther from its position for the original polyelectrolyte-coated particles. With increasing concentration of HP from 10 to 80%, the values of negative zeta potentials increase gradually for both naked and polyelectrolyte-coated particles. The latter shows that protein corona changes gradually in parallel with the increase in hydrodynamic sizes and, seemingly, does not reach saturation, even at the highest concentrations.

### 3.7. MRI Contrast Efficiency of P(PEGMA-AA)@MNPs

We have observed [31] the absence of non-specific binding of P(PEGMA-AA)@MNPs to HeLa cells. The lack of cellular binding or uptake allows the MNPs to flow freely in the vascular system during the course of MRI diagnosis. In addition, specific targeting function can safely be added to the particles, as non-specific binding mechanisms could not compromise their action.

**Figure 12:**
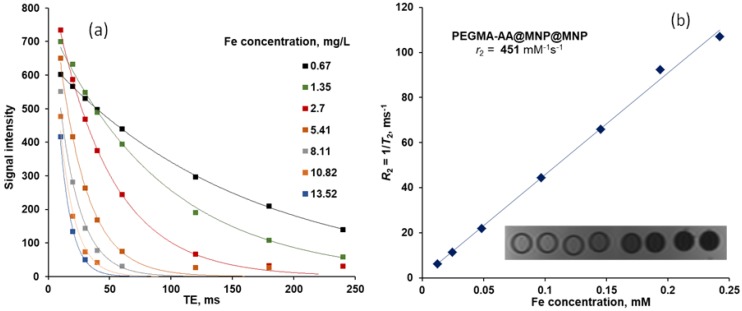
Transverse relaxation curves of P(PEGMA-AA)@MNPs in phantom water measured at 20 °C at increasing iron concentrations (**a**) and the plot for *r*2 relaxivity vs. iron concentration (**b**). The goodness of the linear plot is *R*^2^ = 0.9971. In the inset of the right panel, the T2-weighted image of the samples is shown (measured at TR = 3000 ms repetition time and TE = 40 ms echo delay time) with increasing Fe concentrations.

The MRI contrast efficiency [47,48] of the P(PEGMA-AA)@MNPs was measured at Fe concentrations between 0.5 and 13 mg/L. The iron content was corrected by using the accurate values from ICP measurements. The experimental *r*2 relaxivity, 451 mM^−1^s^−1^ (Figure 12), is one of the highest among published data for core–shell magnetite nanoparticles of similar size [49,50,51].

## 4. Conclusions

The molecular background of post-coating of MNPs with P(PEGMA-AA) brush polyelectrolyte, colloidal stability, pH and salt concentration tolerance of the core–shell MNP product, its hemocompatibility, and MRI contrast efficiency, were studied. Blood sedimentation rate, blood smear, white blood cell viability experiments, and interaction tests of MNP with human plasma proved the hemocompatibility of the PEGylated MNPs. The MRI contrast enhancement of our novel product is noteworthy; the value of the *r*2 relaxivity is one of the highest among the data published in the literature. This fact, together with the magnetic hyperthermia experiments published previously [31], demonstrated the theranostic potential of this newly developed PEGylated MNP product. The high *r*2 relaxivity value and previous hyperthermia analysis [31] both indicate the presence of MNP clusters that have already been observed in dynamic light scattering experiments. We are convinced that our idea to prepare designed PEGylated MNPs via the spontaneous process of self-organization under mild conditions, i.e., the post-coating of magnetic nanocore with multifunctional polyelectrolyte, and optimization for potential biomedical applications, has great advantages, aiming at further scaling-up of the process.

## Figures and Tables

**Table 1:** Characteristics of compounds used for coating magnetite nanoparticles (MNPs).

Coating Material	M_n_ (g/mol)	COOH Functional Groups * (mmol/g)	Molecular Structure
Acrylic acid (AA) small molecule	72	14	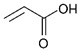
Polyacrylic acid (PAA) homopolymer	1800	14	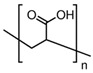
P(PEGMA) comb-like homopolymer	12,400	0	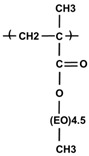
P(PEGMA-AA) comb-like copolymer	6200	5	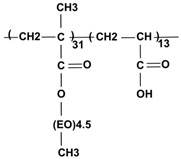

* The amount of carboxylic groups was calculated on the basis of monomer/polymer composition.

## Supplementary Materials

The following are available online at http://www.mdpi.com/2079-4991/8/10/776/s1, “Potentiometric Acid–Base Titration of Polyelectrolytes”, “Additional information on the FT-IR ATR spectra”, Figure S1: FT-IR spectral shifts of AA adsorbed on MNP surface in comparison to the spectra of AA and naked MNP, Figure S2: FT-IR spectra of MNP, P(PEGMA-AA)@MNP and P(PEGMA)@MNP in the ranges of 4000–2400 (a) and 800–500 (b) cm^−1^. For comparison, the PAA@MNP absorption spectrum [37] is also included in (b). The samples were dried from pH ~ 6.5, *I* = 10 mM medium on the ATR crystal, “Additional discussion on the changes in zeta potential due to P(PEGMA-AA) addition”, Figure S3: Zeta potentials of coated nanomagnets in function of P(PEGMA-AA) (red), PAA (yellow) [37], P(PEGMA) (green) and AA (blue) loadings calculated on polymer mass basis (0–0.5 g/g). The measurements are done at pH ~ 6.5 and *I* = 10 mM (NaCl), Figure S4: The pH-dependent mean sizes of P(PEGMA-AA) coated nanomagnets at 0, 0.18, 0.55 and 2.2 mmol COOH/g MNP loadings at 10 mM NaCl. The error bars are omitted for clarity and the lines are drawn to guide the eyes, Figure S5: Evolution of protein corona on P(PEGMA-AA)@MNPs in human plasma in the total measured HP concentration range, as expressed through the changes in average hydrodynamic diameter (*Z*_ave_) measured in dynamic light scattering experiments.
