# Supplementary material for: PEGylation of Superparamagnetic Iron Oxide Nanoparticles with Self-Organizing Polyacrylate-PEG Brushes for Contrast Enhancement in MRI Diagnosis
Source: Nanomaterials (Basel). 2018 Sep 29;8(10):776. doi: 10.3390/nano8100776 (PMC6215243; doi:10.3390/nano8100776)
Supplement: Supplementary file 1 [file nanomaterials-08-00776-s001.pdf]

nanomaterials

PEGylation of Superparamagnetic Iron Oxide Nanoparticles with Self-Organizing Polyacrylate-PEG Brushes for Contrast Enhancement in MRI Diagnosis

Authors: Erzsébet Illés*, Márta Szekeres, Ildikó Y. Tóth, Katalin Farkas, Imre Földesi, Ákos Szabó, Béla Iván, Etelka Tombácz*

Potentiometric acid-base titration of polyelectrolytes

Potentiometric acid–base titration is an adequate analytical tool to determine the actual amount of pH-dependent dissociable groups. Polyelectrolytes with carboxylic moieties acquire charge due to the dissociation of carboxyl groups depending on pH and ionic strength. The net proton excess (on polyelectrolyte molecules) vs. pH function calculated from acid/base titrations reveals the H^+ association/dissociation feature of a polyelectrolyte; namely, the sign of the net proton excess is the same as the charge of polyions and its absolute value is proportional to the amount of protonated/deprotonated groups. In the case of carboxylated compounds, only deprotonation reaction takes place and so the sign of the net proton excess is negative and its absolute values at each pH are equal to the amounts of dissociated carboxyl groups.

Additional information on the FT-IR ATR spectra

The inertness of the acrylic carboxylates of P(PEGMA-AA) in the adsorption on MNPs is in contrast with the behavior of the carboxylates of small molecular AA (Fig. S1). Although the adsorption of AA occurs only to the extent of full surface charge compensation of the magnetite (i.e., 0.06 mmol/g, see the adsorption isotherm, Fig. 2 in the main text), according to FT-IR measurements it affects all the important functional groups and not only the carboxylates (Fig. S1). The shifts in both the C=C stretching (1636 cm^{-1}) and asymmetric and symmetric stretching vibrations of the carboxylates (1545 and 1362 cm^{-1}, respectively) and the C–H bending vibrations of the CH_2/CH_3 groups indicate that the small AA molecules, unlike P(PEGMA-AA), become closely contacted with the MNP surface with practically all functionalities.

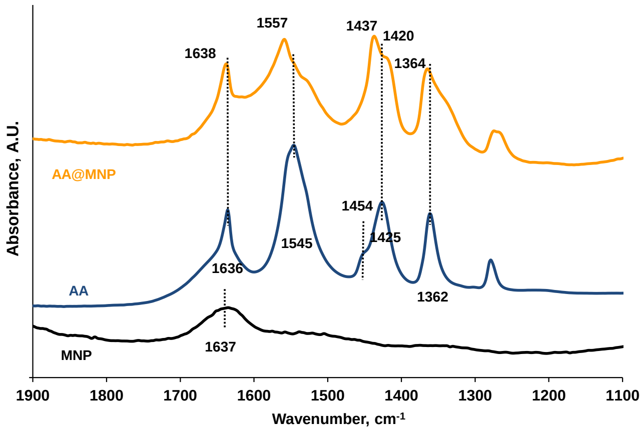

Figure S1. FT-IR spectral shifts of AA adsorbed on MNP surface in comparison to the spectra of AA and naked MNP.

The O–H stretching band of MNP at 3394 cm^{-1} became blue-shifted to 3404 cm^{-1} due to the adsorption of both P(PEGMA) and P(PEGMA-AA) (Fig. S2.a) indicating the increase in the bond

strength because of the change in the environment of ≡Fe–OH from pure aqueous to the polymer coating shell. The absorption bands due to the asymmetric and symmetric C–H stretching modes of methyl and ethyl groups appear clearly in the spectra of coated MNPs in the 2950–2850 cm^{-1} range. In addition, one of the Fe–O stretching bands of magnetite (555 cm^{-1}) is also blue-shifted due to the polymer/polyelectrolyte adsorption to the 560 cm^{-1} region, similarly to that in PAA adsorption (Fig. S2.b).

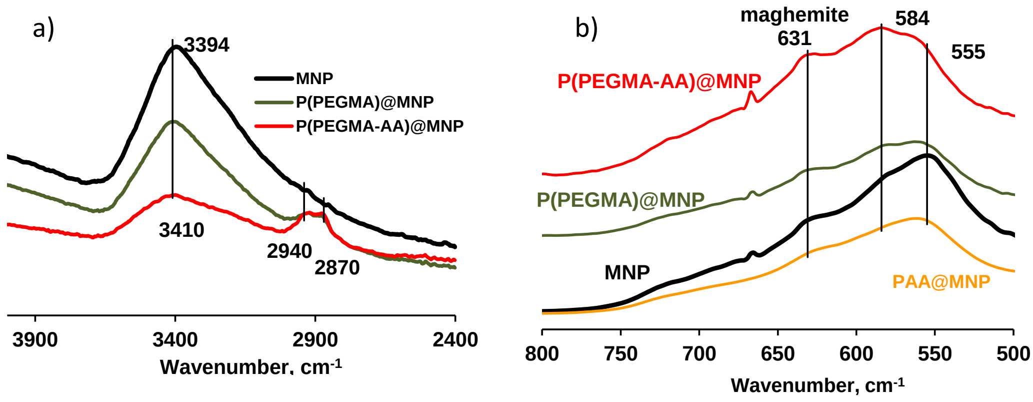

Figure S2. FT-IR spectra of MNP, P(PEGMA-AA)@MNP and P(PEGMA)@MNP in the ranges of 4000–2400 (a) and 800–500 (b) cm^{-1}. For comparison, the PAA@MNP absorption spectrum [37] is also included in (b). The samples were dried from pH ~6.5, I = 10 mM medium on the ATR crystal.

Additional discussion on the changes in zeta potential due to P(PEGMA-AA) addition

In extremely diluted dispersions applied in light scattering (and zeta potential) measurements, the added amounts of carboxylates correspond to the adsorbed amounts as well. Thus, Fig. 4 (main text) represents the effect of adsorbed −COOH units. The adsorption of AA is clearly unable to reverse the MNPs' charge, which is connected with the fact that the carboxylate groups of AA adsorb individually. In contrast, if the carboxylate groups are adsorbed as polyelectrolytes, every attached carboxylate unit brings a multitude of unattached charged moieties to the interface. The inability of the AA anions even to neutralize the MNP charge at the maximum adsorbed amounts can be due to the replacement of uncharged surface ≡Fe–OH sites by also uncharged surface complexes ≡Fe–O–C(O)–R in a ligand exchange adsorption mechanism of the AA (i.e., ≡Fe–OH + R–COO$^-$ = ≡Fe–O–C(O)–R + OH$^-$). Similar adsorption mechanism was verified for poly(acrylic-*co*-maleic acid) (PAM) adsorption on the MNPs [38]. As expected, the addition of uncharged P(PEGMA) also does not change the zeta potential of MNPs, albeit adsorbing via H-bonded structures as demonstrated by the FT-IR ATR spectra above. In addition to ligand exchange, AA can also be adsorbed due to ion-pair formation involving charge compensation. In the latter process, however, the surface charge adjustment mechanism (commonly known for oxide surfaces [S1]) can oppose the charge neutralization of MNPs at AA loadings below 0.06 g/g (0.82 mmol/g) MNP.

[S1]. Lee, E.M.; Koopal, L.K. Adsorption of cationic and anionic surfactants on metal oxide surfaces: Surface charge adjustment and competition effects. J. Colloid Interface Sci. **1996**, 177, 478–489.

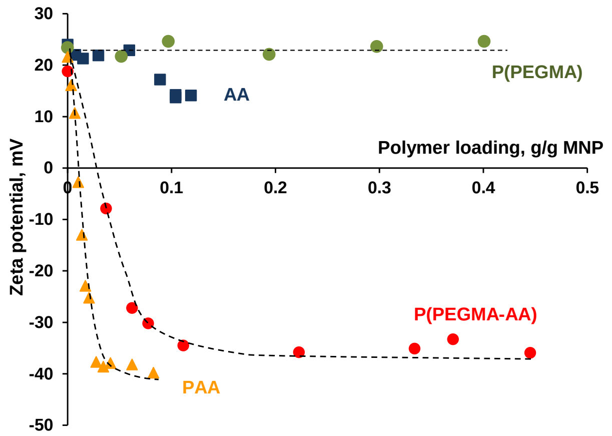

Figure S3. Zeta potentials of coated nanomagnets in function of P(PEGMA-AA) (red), PAA (yellow) [37], P(PEGMA) (green) and AA (blue) loadings calculated on polymer mass basis (0–0.5 g/g). The measurements are done at pH ~6.5 and I = 10 mM (NaCl).

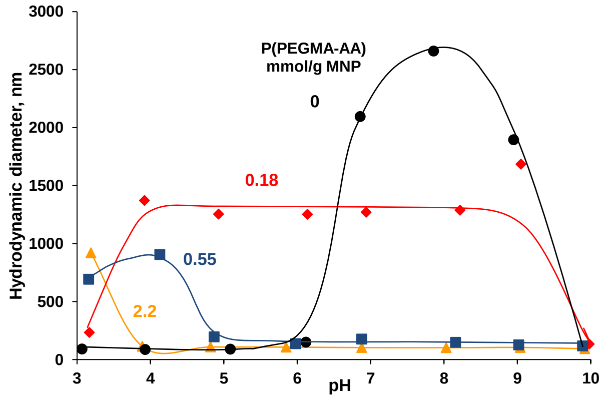

Figure S4. The pH-dependent mean sizes of P(PEGMA-AA) coated nanomagnets at 0, 0.18, 0.55 and 2.2 mmol COOH/g MNP loadings at 10 mM NaCl. The error bars are omitted for clarity and the lines are drawn to guide the eyes.

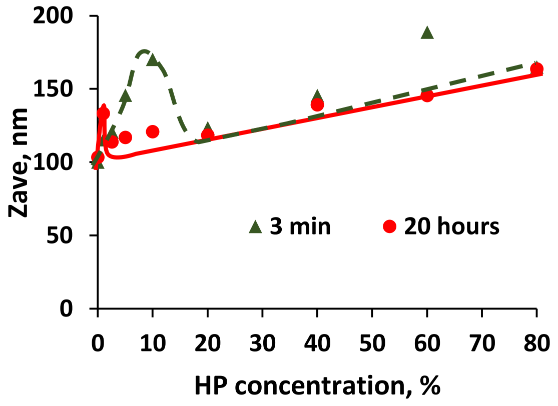

Figure S5. Evolution of protein corona on P(PEGMA-AA)@MNPs in human plasma in the total measured HP concentration range, as expressed through the changes in average hydrodynamic diameter (Z_{ave}) measured in dynamic light scattering experiments.
